# Circular RNA-DPP4 serves an oncogenic role in prostate cancer progression through regulating miR-195/cyclin D1 axis

**DOI:** 10.1186/s12935-021-02062-z

**Published:** 2021-07-16

**Authors:** Deping Yang, Bo Yang, Yanjun Zhu, Qianlin Xia, Yan Zhang, Xin Zhu, Jianming Guo, Tao Ding, Jianghua Zheng

**Affiliations:** 1 Department of Laboratory Medicine, Shanghai University of Medicine & Health Sciences Affiliated Zhoupu Hospital, Shanghai, 201318 China; 2 Department of Urology, Shanghai University of Medicine & Health Sciences Affiliated Zhoupu Hospital, Shanghai, 201318 China; 3 Department of Urology, Zhongshan Hospital, Fudan University, Shanghai, 200032 China; 4 Department of Laboratory Medicine, The Sixth People’s Hospital East Campus, Shanghai Jiao Tong University, Shanghai, 201306 China; 5 Department of Urology, The Sixth People’s Hospital South Campus, Shanghai Jiao Tong University, Shanghai, 201489 China

**Keywords:** Prostate cancer, Circular RNA DPP4 (circDPP4), microRNA-195, Cell cycle, Cyclin D1

## Abstract

**Background:**

Recently, more and more studies have highlighted the critical regulatory roles of circular RNAs (circRNAs), a class of non-coding RNAs, in the progression of many human cancers, including prostate cancer (PCa). circRNA microarray analysis was performed to identify circRNAs that are differentially expressed in PCa tissues.

**Methods:**

104 pairs of PCa tissues and matched adjacent normal prostate tissues (at least 2 cm distal to the tumor margin) were obtained. circRNA microarray analysis was performed on four pairs of PCa tissues and matched adjacent normal prostate tissues to investigate the potential involvement of circRNAs in PCa. Flow cytometric analysis was performed to investigate whether the effect of circDPP4 on PCa cell proliferation was associated with the alteration in cell cycle progression. The role of circDPP4 in PCa tumor growth was further explored in vivo.

**Results:**

We found that circDPP4 was overexpressed in PCa tissues and cell lines, and its expression was closely associated with Gleason score and clinical stage of PCa patients. In vitro loss- and gain-of-function experiments demonstrated that circDPP4 knockdown inhibited, whereas circDPP4 overexpression promoted the proliferation, migration, invasion and cell cycle progression of PCa cells. Knockdown of circDPP4 also suppressed PCa tumor growth in vivo. We further found that circDPP4 functioned as a competing endogenous RNA (ceRNA) for miR-195 in PCa cells, and miR-195 negatively regulated the expression of oncogenic cyclin D1. Rescue experiments suggested that restoration of miR-195 blocked the oncogenic role of circDPP4 in PCa cells.

**Conclusions:**

Taken together, our findings revealed a novel regulatory mechanism between circDPP4 and miR-195/cyclin D1 axis, and offered novel strategies for the treatment of PCa.

**Supplementary Information:**

The online version contains supplementary material available at 10.1186/s12935-021-02062-z.

## Background

Globally, prostate cancer (PCa) is the second most frequent cancer and the fifth leading cause of cancer-related deaths in male [1]. Over the last few years, the incidence and mortality rates of PCa have increased rapidly in China, including Shanghai [2, 3]. Despite the fact that great progress has been made in the diagnostic and therapeutic methods, the management of PCa still remains one of the most serious medical challenges. Accordingly, getting a better understanding of the potential carcinogenetic mechanisms is of critical importance to identify novel diagnostic and therapeutic targets for PCa patients.

Circular RNAs (circRNAs), a large group of non-coding RNAs, are characterized by their covalently closed loop structures without a 5' cap or a 3′ poly A tail [4]. circRNAs are abundant in the human transcriptome, and compared with linear RNAs, circRNAs are more stable and conserved in sequence. The term “circRNA” was first proposed by Sanger in 1976 [5], and in recent years, emerging evidence has shown the biological roles of circRNAs in multiple processes such as cell growth, migration and invasion, which are thought to be critical for the tumorigenesis and progression of various human cancers [6, 7]. Mechanically, circRNAs function as microRNA (miRNA) sponges, RNA-binding proteins and transcriptional regulators, involving the pathogenesis of these malignancies [8, 9]. Many studies demonstrated that circRNAs is mainly involved in the regulation of gene expression and specific biological functions by sponging miRNAs. For instance, circRNA CDR1 was reported as a sponge of miR-432-5p to regulate expression of E2F3, a direct target gene of miR-432-5p, and promote proliferation, migration and invasion of PCa cells [10]. circANKS1B plays an oncogenic role in the progression of PCa via sponging miR-152-3p and upregulating TGF-α expression [11].

As an important regulator of cell cycle, cyclin D1 is involved in the transition from G1 to S phase by regulating cyclin-dependent kinase (CDK)4 and CDK6 [12]. Dysregulation of cyclin D1 expression drives uncontrolled cell proliferation and results in the development of human cancers. It has been reported that the expression level of cyclin D1 was markedly upregulated in human PCa, breast cancer, non-small cell lung cancer and hepatocellular carcinoma [13–16]. Several studies have shown that cyclin D1 expression could be negatively regulated by miR-195 to affect the tumorigenicity and invasiveness of laryngeal squamous cell carcinoma and glioblastoma cells [17, 18]. Recently, Duan et al. reported the biological roles of circMYLK/miR-195/cyclin D1 axis in the development and progression of laryngeal squamous cell carcinoma, providing a novel therapeutic target for these patients [17]. However, the relationship between circRNAs and miR-195/cyclin D1 axis in the pathogenesis of PCa remains unclear.

In the present study, we identified a novel circRNA circDPP4, which is significantly upregulated in PCa tissues, by human circRNA microarray analysis. Subsequently, we performed a series of functional assays and molecular mechanism analysis to investigate the effect of its overexpression on biological characteristics of PCa cells and determine whether circDPP4 functions as an oncogene to promote the development and progression of PCa via sponging miR-195 and inducing cyclin D1 overexpression.

## Methods

### Clinical specimens

104 pairs of PCa tissues and matched adjacent normal prostate tissues (at least 2 cm distal to the tumor margin) were obtained from patients who were diagnosed with PCa by histopathology and undergone surgery at Shanghai University of Medicine & Health Sciences Affiliated Zhoupu Hospital. In this study, early stage PCa was defined as a tumor with ≤ stage cT2a–T2c without the evidence of metastases; otherwise, advanced stage PCa was defined. The clinicopathological data of these patients are listed in Additional file 4: Table S1. All patients did not receive any other treatment prior to operation. After removal from the body, tissue samples were snap-frozen in liquid nitrogen and stored at − 80 °C. The use of human tissues was approved by the Ethics Committee of Shanghai University of Medicine & Health Sciences Affiliated Zhoupu Hospital, and all patients or their relatives signed the informed consent.

### CircRNA microarray analysis

A total of 4 pair matched PCa and adjacent normal tissues were used for microarray analysis to identify differentially expressed circRNAs involved in tumorigenesis and progression of PCa as previously described [19]. Total RNA was extracted using TRIzol reagent (Invitrogen, Carlsbad, CA, USA). RNA concentration and quality were analyzed using Agilent Bioanalyzer 2100 (Agilent Technologies, Santa Clara, CA, USA). Then, RNA samples were amplified, labeled using Low Input Quick Amp WT Labeling Kit (Agilent Technologies), and hybridized onto SBC-ceRNA (4 * 180 K) (BH170234; Shanghai Bohao Biotechnology co., LTD, Shanghai, China). The hybridized microarrays were washed and then scanned with Agilent Microarray Scanner (Agilent Technologies). Quantile normalization and subsequent data processing were performed using the R software limma package. |log_2_ fold change| > 1.0 and *P* value < 0.05 were considered significant. Hierarchical clustering analysis was performed to show the differentially expressed circRNAs among samples.

### Cell culture and transfection

Four human PCa cell lines (PC3, DU145, LNCaP and 22RV1), a human prostate epithelial cell line (RWPE1) and human embryonic kidney cell line (HEK293T) purchased from American Type Culture Collection (Manassas, VA, USA) were cultured in RPMI 1640 medium (Invitrogen) containing 10% fetal bovine serum (FBS; Biowest, Loire, France), 100 U/ml penicillin sodium and 100 mg/ml streptomycin sulfate at 37 °C in an atmosphere containing 5% CO_2_. The cells were periodically confirmed negative for mycoplasma contamination and were authenticated by DNA profiling.

MiR-195 mimics, miR-195 inhibitor, mimics control and inhibitor control were chemically synthesized by GenePharma Co. Ltd. (Shanghai, China). The synthesized circDPP4 gene fragment was inserted into the pcDNA3.1 vector (Invitrogen) to construct overexpression vector. An empty pcDNA3.1 vector was used as a negative control. Short-hairpin RNA oligo 5′-UGUUCUUCUUGUUUGACAGGA-3′) directed against circDPP4 was synthesized and inserted into the SuperSilencing shRNA plasmid pGPU6/Neo (GenePharma Co. Ltd.). Cells at 70–80% confluence were used for transfection using Lipofectamine 2000 (Invitrogen).

### RNA extraction and RT-qPCR analysis

The isolated RNA was reverse transcribed into cDNA by using the PrimeScript™ RT reagent kit (TaKaRa, Dalian, China). qPCR reactions were then carried out on an ABI PRISM 7500 fast Sequence Detection System (Applied Biosystems, Foster City, CA, USA) using the SYBR Premix Ex Taq II kit (TaKaRa). The 2^−ΔΔCt^ method was used to determine relative expression levels of crircDPP4 and miR-195 in PCa tissues and cell lines [20]. GAPDH was chosen as the internal control for circRNA and mRNA. U6 was chosen as the internal control for miRNA. The primer sequences of circDPP4 and GAPDH are crircDPP4 forward: 5′-AATGAGAGGGAAGAGCGGAG-3′; crircDPP4 reverse: 5′-ACATCCACGTCCTTTCCCAT-3′; GAPDH forward: 5′-CACATCGCTC AGACACCATG-3′; GAPDH reverse: 5′-TGACGGTGCCATGGAA TTTG-3′. Other primer sequences for RT-qPCR assay are presented in Additional file 4: Table S1.

### CCK-8 assay

Cell proliferation was detected using Cell Counting Kit-8 (CCK-8; Dojindo Laboratories, Kumamoto, Japan). Cells were seeded at a density of 2000 cells/well in 96-well plates. 10 μl of CCK-8 solution was added into each well, and the plants were incubated for another 2 h. The absorbance was measured with a microplate reader (Dynex, Chantilly, VA, USA).

### EdU staining assay

Cells were cultured in 24-well plates, and 10 μM of EdU reagent (RiboBio, Guangzhou, China) was added to each well according to the manufacturer’s protocol. After 2 h of incubation at 37 °C, the cells were fixed with 4% paraformaldehyde for 30 min, and then the nuclei were stained with Hoechst 33,342 for 5 min. Cells were visualized under a fluorescent microscope (Olympus, Tokyo, Japan) and the ratio of EdU-positive cells to total cells was calculated.

### Transwell assay

2 × 10^5^ cells were resuspended in 100 µl of medium and seeded into the upper chambers of Transwell plates (8-µm pore size; Corning-Costar, Cambridge, MA, USA) coated with or without Matrigel (Corning-Costar) and containing 600 μl of medium supplemented with 10% FBS in the lower chambers. Following 24 h of incubation, the cells on the upper membrane surface were carefully scraped off with cotton swabs, and the cells on the lower membrane surface were fixed with 4% paraformaldehyde, stained with 0.1% crystal violet and photographed under a light microscope. Five random fields were counted per chamber.

### Cell cycle analysis

The Cycle TEST PLUS DNA Reagent Kit (BD Biosciences, San Jose, CA, USA) was used to detect cell cycle progression. The cells were collected and fixed in 75% ethanol overnight at 4 °C. Then the fixed cells were incubated with 100 µl RNase A reagent (Keygen Biotech, Nanjing, China) for 30 min at 37 °C, and stained with 20 μg/ml PI for 10 min at room temperature. The percentage of cells in G0/G1, S, and G2/M phases was analyzed by flow cytometry (BD Biosciences).

### Western blot analysis

Total protein was extract from tissues and cells using RIPA lysis buffer (Beyotime, Shanghai, China) and quantified by a BCA Protein Assay Kit (Solarbio, Beijing, China). Equivalent amount of proteins were separated by SDS-polyacrylamide gel electrophoresis and transferred onto PVDF membranes (Millipore, Billerica, MA, USA). The membranes were blocked with 5% skimmed milk and probed with specific primary antibodies against cyclin D1 (1:500; Proteintech, Chicago, IL, USA) and GAPDH (1:1000; Cell Signaling Technology, Danvers, MA, USA), followed by incubation with appropriate HRP-linked secondary antibodies (1:1000; Beyotime) for 1 h at room temperature. The bands were visualized using an enhanced chemiluminescence kit (Santa Cruz, Dallas, TX, USA). To ensure equal protein loading, GAPDH was considered as the loading control.

### Subcellular fractionation location

The PARIS Kit (Invitrogen) was used to separate the nuclear and cytoplasmic fractions of PCa cells. RT-qPCR analysis was performed to detect the expression levels of circDPP4, GAPDH and U6 in the nuclear and cytoplasmic fractions. GAPDH was used as the cytoplasmic control, and U6 was used as the nuclear control.

### Fluorescence in situ hybridization (FISH) assay

The localization of circDPP4 and miR-195 in PCa cells was detected by fluorescence in situ hybridization (FISH) kit (ThermoFishers, USA). PC3 cells were harvested and fixed with 4% paraformaldehyde (PFA) for 15 min at room temperature. The fluorescein-labeled probes specific for circDPP4 (green fluorescence) and miR-195 (red fluorescence) were hybridized at 37 °C overnight. Moreover, cell nuclei (blue fluorescence) were stained with 4′,6-diamidino-2-phenylindole (DAPI). All procedures were performed according to the manufacturer’s ViewRNA ISH Assays protocol. A confocal microscopy was used to observe the presence of circDPP4 and miR-195 in PCa cells.

### Dual-luciferase reporter assay

The fragment from circDPP4 or cyclin D1 mRNA 3′-UTR containing the predicted miR-195 binding site was amplified by PCR and cloned into psiCHECK-2 luciferase reporter vector (Promega, Madison, WI, USA). circDPP4-MUT and cyclin D1-MUT reporters were generated using Quickchange XL site-directed mutagenesis kit (Agilent Technologies). All constructs were confirmed by DNA sequencing. PC3 cells at 80% confluence in 96-well plate were co-transfected with the luciferase reporter vectors and miR-195 mimics or mimics control using Lipofectamine 2000. After 48 h of transfection, luciferase activity was measured using the Dual-Luciferase Reporter Assay system (Promega).

### RNA-pull down assay

The biotin-labeled probe with wild type miR-195 (biotin-wt-miR-195) and mutant type miR-195 (biotin-mut-miR-195) and negative control (biotin-NC) were synthesized. Then, they were transfected into PC3 and LNCaP cells. After 48 h, the cells were harvested, lysed by lysis buffer and incubated with streptavidin beads for 2 h at 4 °C. Next, the beads were washed by lysis buffer for three times, and the RNA complex was purified with TRIzol reagent. qRT-PCR analysis was conducted to detect the abundance of circDPP4 in PCa cells.

### Animal tumor xenograft model

Twelve male nude mice (athymic, Balb/c nu/nu, aged 5–6 weeks, weighting 18–20 g) were purchased from the Slac Laboratory Animal Center (Shanghai, China) and kept under specific pathogen-free conditions. 1 × 10^6^ PC3 cells stably transfected with sh-circDPP4 or sh-NC were resuspended in 200 μl of PBS and inoculated subcutaneously into a single side of the posterior flank of nude mice. Six mice were included in each group. Tumors were measured using a caliper every week and tumor volume was calculated following the formula: V = 0.5 × D × d^2^ (V, volume; D, longitudinal diameter; d, latitudinal diameter). Five weeks after cell inoculation, mice were sacrificed by CO_2_ asphyxiation, and the xenograft tumors were excised, weighted and subjected to H&E staining and immunohistochemistry (IHC) analysis of Ki-67 protein.

All experimental procedures involving the use of animals were approved by the Ethics Committee of Zhongshan Hospital, and all efforts were made to minimize animal suffering, according to the NIH Guide for the Care and Use of Laboratory Animals.

### Statistical analysis

All data were analyzed by GraphPad Prism 6.0 software (GraphPad Software, San Diego, CA, USA) and SPSS 16.0 software (SPSS, Chicago, IL, USA). Comparisons between groups were analyzed using the Student’s *t*-test and one-way analysis of variance or χ^2^ test, as appropriate. Correlation between circDPP4 and miR-195 expression in PCa tissues was evaluated by Pearson’s correlation analysis. *P* < 0.05 was considered to indicate a statistically significant difference. Original images were included in Additional file 6.

## Results

### CircDPP4 is upregulated in human PCa tissues and significantly correlated with the disease progression

First, circRNA microarray analysis was performed in four pairs of PCa tissues and matched adjacent normal prostate tissues to investigate the potential involvement of circRNAs in PCa. The relevant findings were previously described [19]. Based on the threshold of |log2 fold change| > 2.0 and adjusted P < 0.05, a total of 117 circRNAs were significantly upregulated, and 904 circRNAs were downregulated in four PCa tissue samples (Additional file 1: Fig. S1 and Additional file 6).

**Fig. 1:**
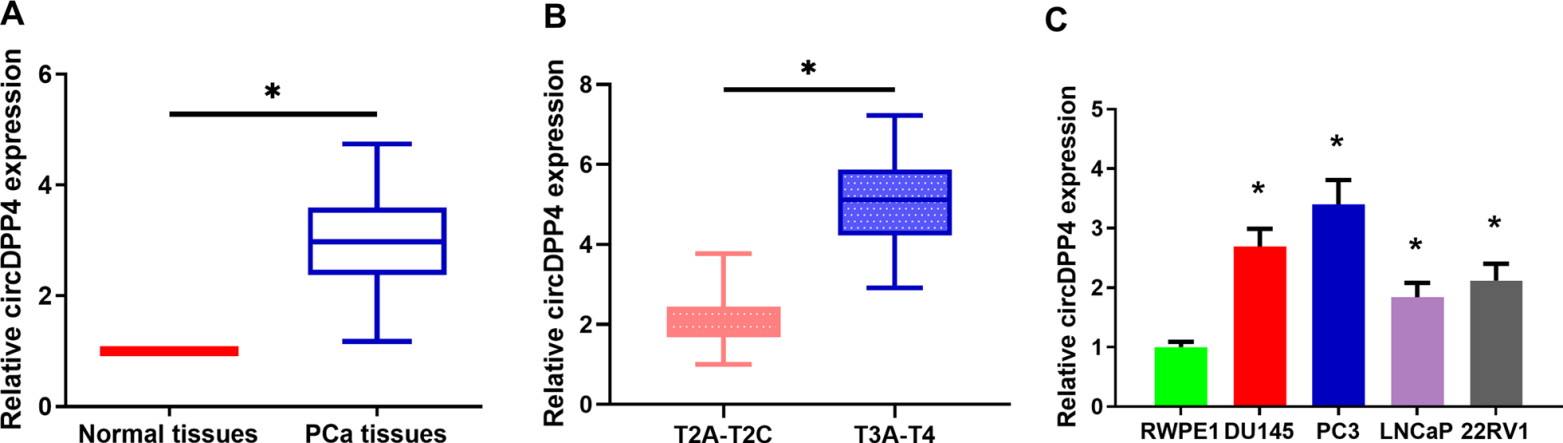
circDPP4 is upregulated in human PCa tissues. **A** RT-qPCR analysis of circDPP4 expression levels in 104 pairs of PCa tissues and adjacent normal tissues. *P < 0.05 vs. normal tissues. **B** Expression of circDPP4, showing higher level in late stage than in early stage of PCa patients. **C** RT-qPCR analysis of circDPP4 expression levels in PCa cell lines and normal counterpart. *P < 0.05 vs*.* normal cells

We found that hsa_circ_0056881, derived from gene DPP4, was among the top upregulated circRNAs. Hsa_circ_0056881 was therefore termed as “circDPP4”. We then validated the expression of circDPP4 in a cohort of 104 pair of PCa tissues and matched adjacent normal tissues using RT-qPCR analysis. As shown in Fig. 1A, the expression of circDPP4 was remarkably increased in PCa tissues than in the matched adjacent normal tissues. Moreover, expression of circDPP4 increased with the severity of disease as it is higher in stage cT3a–T4 PCa than in stage cT2a–T2c (Fig. 1B, P < 0.05). In addition, circDPP4 showed a higher expression in four PCa cell lines (PC3, DU145, LNCaP and 22RV1) compared to normal prostate epithelial cell line (RWPE1) (Fig. 1C).

To further evaluate the clinical significance of circDPP4 in PCa, we explored the correlation between circDPP4 expression and clinicopathological features of PCa patients. The 104 PCa patients were allocated into a high expression group (n = 52; > median) and a low expression group (n = 52; < median) based on the median expression level of circDPP4. As indicated in Additional file 5: Table S2, PCa patients with high circDPP4 expression had a higher Gleason score (P = 0.015) and were at more advanced clinical stage (P = 0.026) than those with low circDPP4 expression.

### CircDPP4 promotes PCa cell proliferation, migration and invasion in vitro

**Fig. 2:**
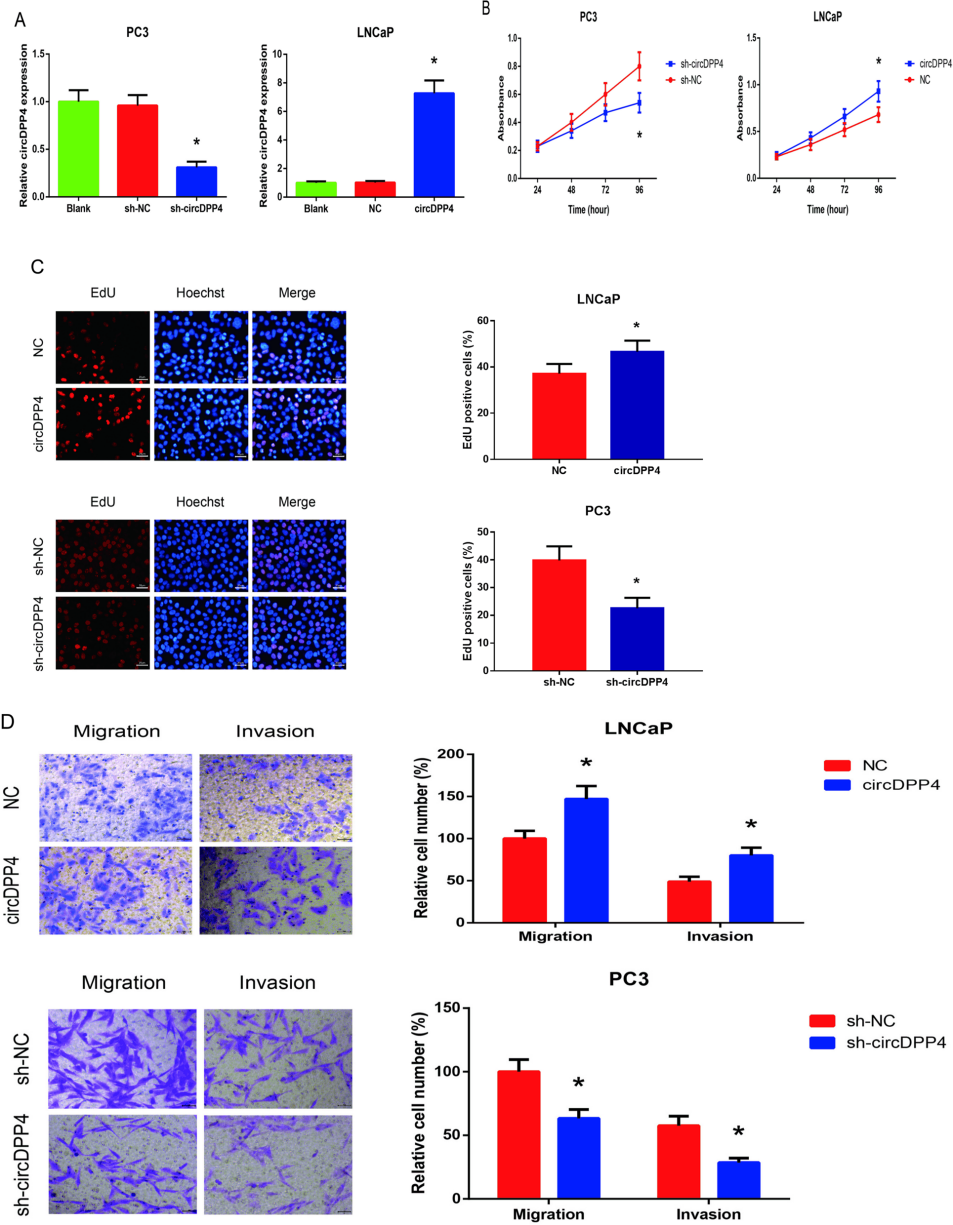
circDPP4 promotes PCa cell proliferation, migration and invasion in vitro. **A** RT-qPCR analysis of circDPP4 expression levels in PC3 and LNCaP cells after transfection. **B** The proliferation of PC3 and LNCaP cells after transfection detected by CCK-8 assay. **C** The proliferation of PC3 and LNCaP cells after transfection detected by EdU staining assay. **D** The migration and invasion of PC3 and LNCaP cells after transfection detected by transwell assay. *P < 0.05 vs*.* sh-NC or empty vector-transfected cells

The increased circDPP4 expression in human PCa samples prompted us to investigate the biological role of circDPP4 in PCa tumorigenesis. circDPP4 was knocked down in PC3 cells by transfection of sh-circDPP4 and was overexpressed in LNCaP cells by transfection of pcDNA3.1-circDPP4. The transfection efficacies were confirmed by RT-qPCR analysis (Fig. 2A). The results of CCK-8 assay showed that proliferation of sh-circDPP4-transfected PC3 cells was notably suppressed, whereas overexpression of circDPP4 enhanced proliferation of LNCaP cells (Fig. 2B). In addition, as indicated by EdU staining assay, circDPP4 knockdown reduced whereas its overexpression increased the proportion of EdU incorporated PCa cells (Fig. 2C). We further evaluated the role of circDPP4 in migration and invasion of PCa cells. The results of Transwell assay revealed that migratory and invasive capacities of PCa cells were significantly weakened by circDPP4 knockdown and remarkably enhanced by circDPP4 overexpression (Fig. 2D).

### CircDPP4 promotes PCa cell cycle progression

**Fig. 3:**
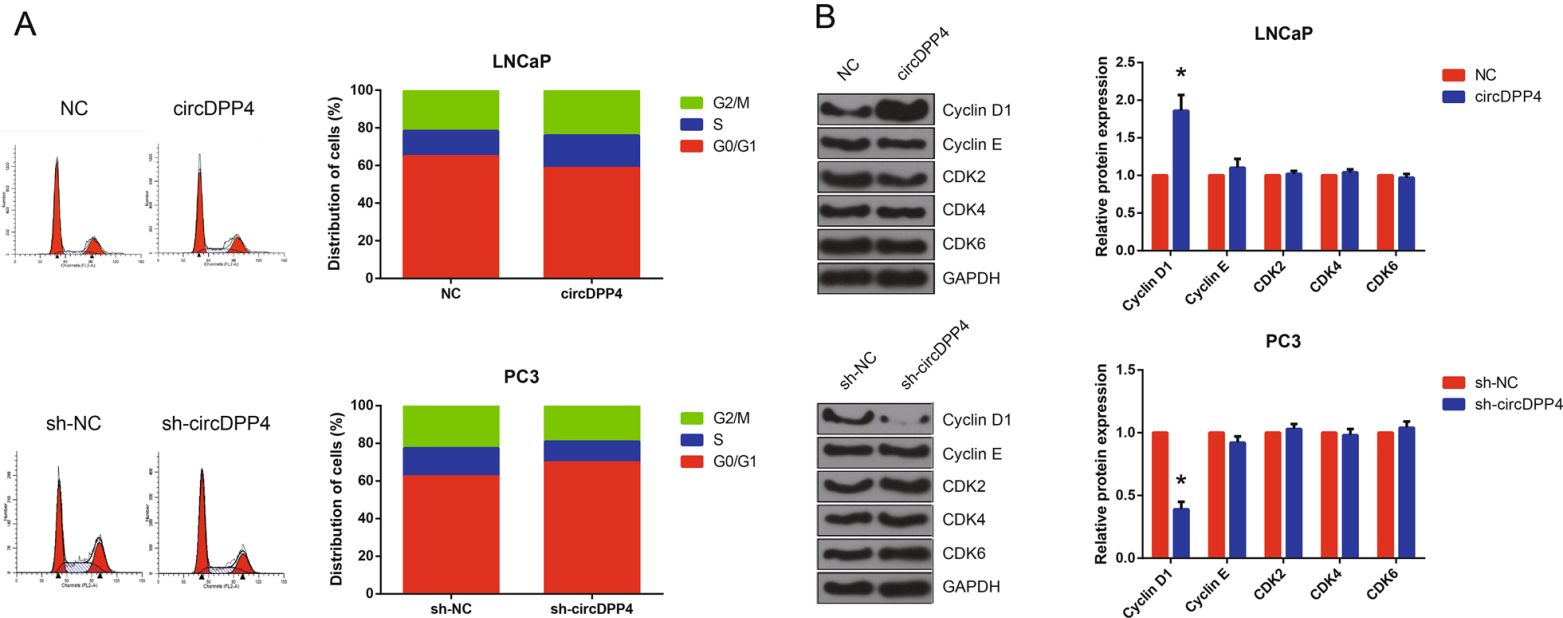
circDPP4 promotes PCa cell cycle progression. **A** The cell cycle distribution of PC3 and LNCaP cells after transfection detected by flow cytometric analysis. **B** Western blot analysis of cell cycle-related protein expression levels in PC3 and LNCaP cells after transfection. *P < 0.05 vs. sh-NC or empty vector-transfected cells

Moreover, flow cytometric analysis was performed to investigate whether the effect of circDPP4 on PCa cell proliferation was associated with the alteration in cell cycle progression. The results indicated that circDPP4 overexpression promoted cell cycle progression of LNCaP cells, whereas the proportion of sh-circDPP4-transfected PC3 cells at G0/G1 phase was notably increased (Fig. 3A). The effects of circDPP4 on cell cycle-related proteins in PCa cells were further evaluated. As shown in Fig. 3B, cyclin D1 was the only up-regulated protein in LNCaP cells with circDPP4 overexpression. On the contrary, circDPP4 knockdown reduced cyclin D1 protein expression in PC3 cells.

### Knockdown of circDPP4 inhibits PCa tumor growth in vivo

**Fig. 4:**
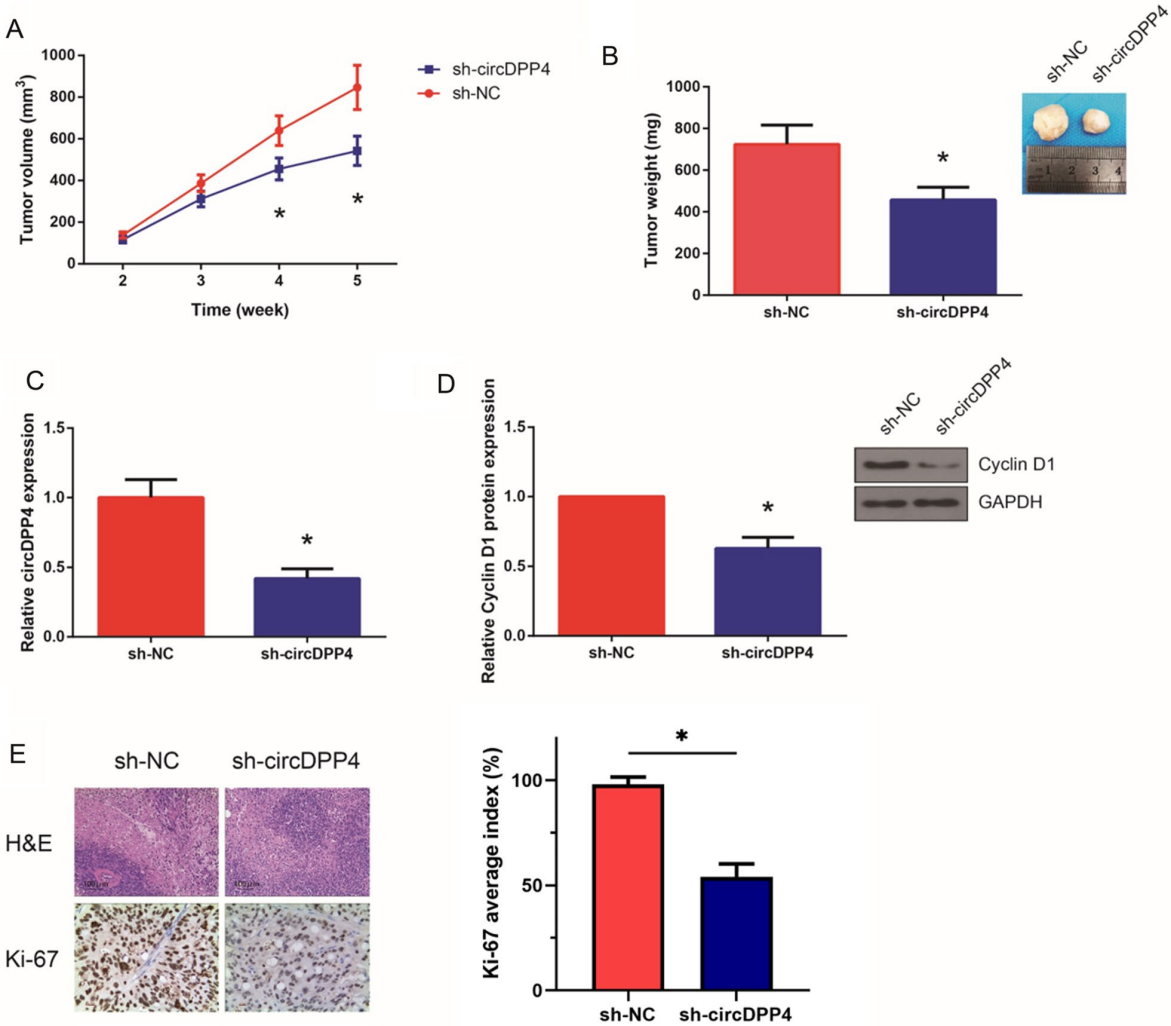
Knockdown of circDPP4 inhibits PCa tumor growth in vivo. **A** Tumor volume was monitored every week. **B** Representative images of tumors and quantification of tumor weights. **C** RT-qPCR analysis of circDPP4 expression levels in the tumor tissues. **D** Western blot analysis of cyclin D1 protein expression levels in the tumor tissues. **E** H&E staining and immunohistochemistry (IHC) analysis of Ki-67 protein in the tumor tissues. *P < 0.05 vs*.* sh-NC group

The role of circDPP4 in PCa tumor growth was further explored in vivo. In accordance with the in vitro findings, knockdown of circDPP4 led to a remarkable attenuation of PCa tumor growth (Fig. 4A). At 5 weeks of post-injection, the average tumor weight of the sh-circDPP41 group was obviously reduced (Fig. 4B), and the downregulation of circDPP4 in the tumors formed from sh-circDPP4-transfected cells was validated by RT-qPCR analysis (Fig. 4C). Moreover, as shown in Fig. 4D, cyclin D1 protein expression was reduced in the tumor tissues from the sh-circDPP4 group. The staining intensity of proliferation antigen Ki-67 in the tumor tissues was also notably reduced by circDPP4 knockdown (Fig. 4E).

### CircDPP4 acts as a ceRNA for miR-195 in PCa cells

**Fig. 5:**
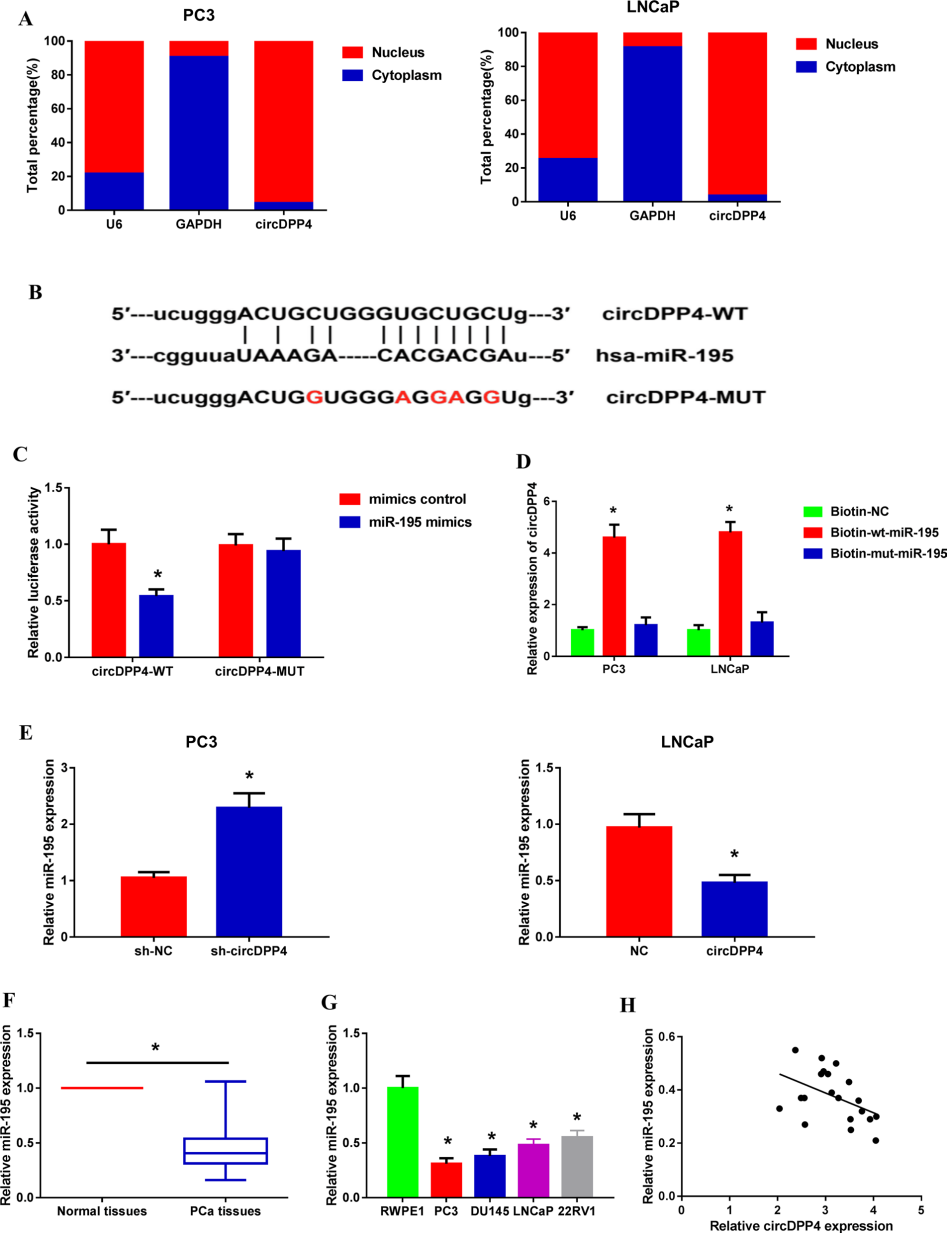
circDPP4 acts as a ceRNA for miR-195 in PCa cells. **A** Relative expression levels of circDPP4 in the nuclear and cytoplasmic fractions of PCa cells. **B** The predicted miR-195 binding sites in circDPP4. **C** Dual-luciferase reporter assay performed to validate the direct binding relationship between circDPP4 and miR-195. *P < 0.05 vs. mimics control-transfected cells. **D** The interaction between circDPP4 and miR-195 in PCa cells was measured by RNA-pull down assay. **E** RT-qPCR analysis of miR-195 expression levels in PC3 and LNCaP cells after transfection. *P < 0.05 vs. sh-NC or empty vector-transfected cells. **F** RT-qPCR analysis of miR-195 expression levels in 104 pairs of PCa tissues and adjacent normal tissues. *P < 0.05 vs*.* normal tissues. **G** RT-qPCR analysis of miR-195 expression levels in PCa cell lines and normal counterpart. *P < 0.05 vs. RWPE1 cells. **H** The inverse correlation between circDPP4 and miR-195 expression in PCa tissues of 20 randomly selected patients

We further uncovered that circDPP4 was predominantly localized in the cytoplasmic fraction of PC3 and LNCaP cells (Fig. 5A), indicating that circDPP4 might function as a ceRNA to sequester miRNAs in PCa cells. Through the Starbase database (http://starbase.sysu.edu.cn/index.php), we found that circDPP4 has a binding site for miR-195 (Fig. 5B). To validate the direct binding relationship between circDPP4 and miR-195, dual-luciferase reporter assay was conducted, and the results showed that the luciferase activity was reduced by more than 40% when PC3 cells were co-transfection with circDPP4-WT reporter and miR-195 mimics (Fig. 5C). Moreover, RNA pull-down assay revealed that circDPP4 was significantly enriched with biotin-labeled wt-miR-195 in PCa cells compared with negative control and/or biotin-labeled mut-miR-195. These findings further supported a direct interaction between circDPP4 and miR-195 in PCa cells (Fig. 5D). We also found that miR-195 expression was decreased in circDPP4 overexpressed LNCaP cells, but increased in circDPP4 knockdown PC3 cells (Fig. 5E). Besides, expression of miR-195 was significantly decreased in PCa tissues and cell lines (Fig. 5F, G). MiR-195 expression was inversely correlated with circDPP4 expression in PCa tissues of 20 randomly selected patients (Fig. 5H).

### Cyclin D1 is a downstream target of miR-195 in PCa cells

Bioinformatics analysis also demonstrated that there are two conserved binding sites for miR-195 on the 3′-UTR of cyclin D1 mRNA (Additional file 2: Fig. S2A). In addition, as shown in Additional file 2: Fig. S2B, miR-195 mimics significantly inhibited luciferase activity of cyclin D1-WT reporter but not the MUT reporter in HEK293T cells. Meanwhile, cyclin D1 protein level was decreased by miR-195 mimics in PC3 cells and increased by miR-195 inhibitor in LNCaP cells (Additional file 2: Fig. S2C). Furthermore, we found that miR-195 overexpression suppressed whereas miR-195 inhibition promoted the proliferation of PCa cells (Additional file 2: Fig. S2D).

### MiR-195 blocks the oncogenic role of circDPP4 in PCa cells

**Fig. 6:**
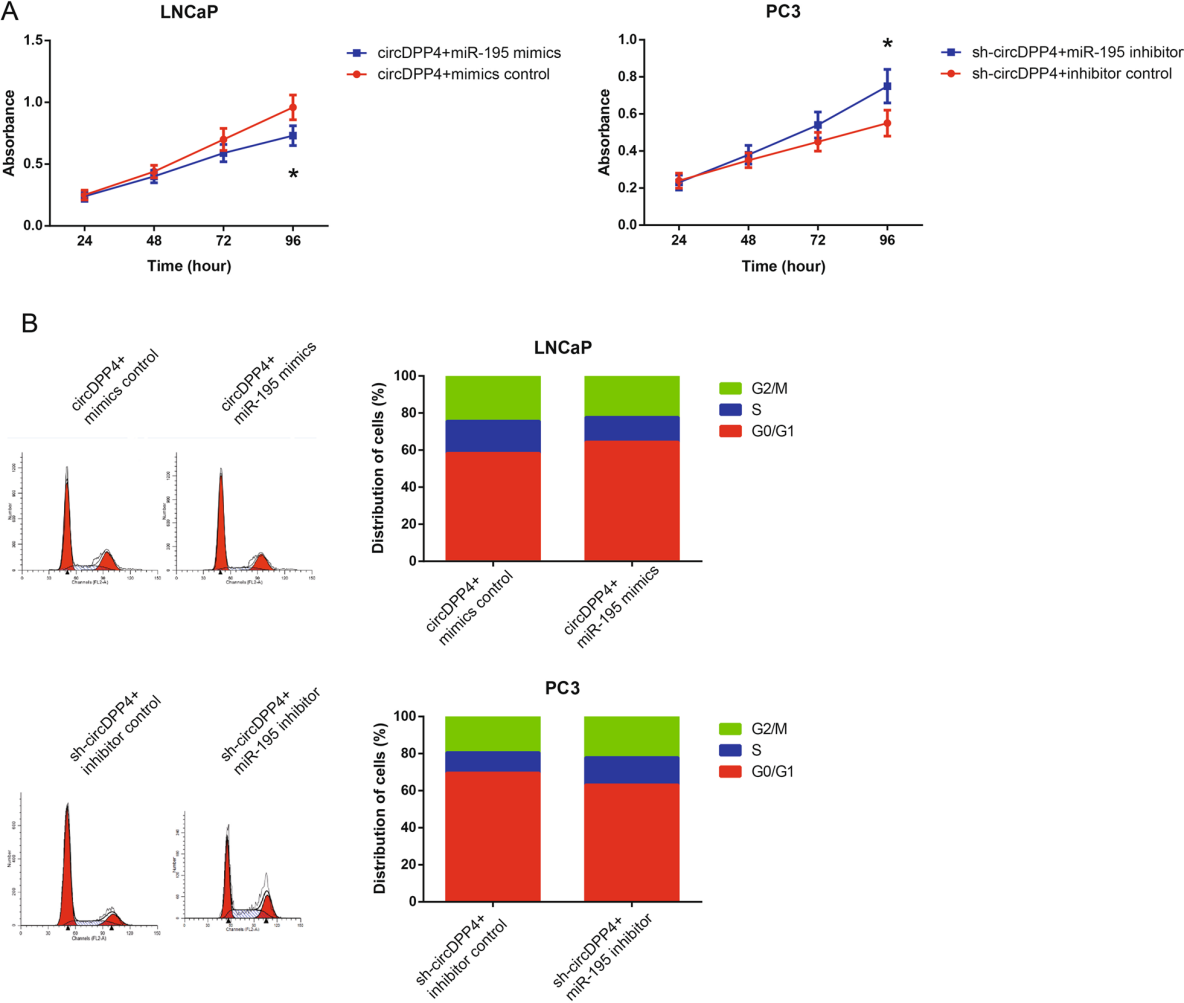
miR-195 blocks the oncogenic role of circDPP4 in PCa cells. **A** Proliferation of PC3 and LNCaP cells after transfection detected by CCK-8 assay. **B** Cell cycle distribution of PC3 and LNCaP cells after transfection detected by flow cytometric analysis. *P < 0.05 vs*.* mimics control or inhibitor control-transfected cells

We then performed the rescue experiments and demonstrated that restoration of miR-195 blocked the effects of circDPP4 overexpression on the proliferation and cell cycle distribution of LNCaP cells (Fig. 6A, B). In contrast, co-transfection with miR-195 inhibitor abolished the effects of circDPP4 knockdown on the proliferation and cell cycle distribution of PC3 cells. Furthermore, we performed RNA-ISH assay to validate the localization of circDPP4 and miR-195 in tumor tissue and PC3 cell line and found that circDPP4 and miR-195 are co-localized (Additional file 3: Fig. S3).

## Discussion

The role of non-coding RNAs including circRNAs has attracted extensive attention recently in the study of cancer biology. Up to now, several circRNAs have been identified to be closely related to PCa, a clinically heterogeneous and multifactorial disease. For example, circMYLK is overexpressed in PCa tissues and PCa cells [21], and upregulated circ-102004 promotes cell proliferation in PCa [22]. Previously we have also identified two PCa-related circRNAs circ_0057558 and circ_0062019 in PCa [19]. We believe circRNA-based therapy will be a feasible approach for PCa treatment.

In this study, through human circRNA microarray analysis followed by RT-qPCR validation, we identified a circRNA derived from DPP4 gene locus, termed as circDPP4 and found its expression was significantly increased in both clinical PCa tissues and PCa cell lines, implying its oncogenic effect. In vitro gain-of-function and loss-of-function experiments showed that circDPP4 overexpression promoted, whereas circDPP4 knockdown suppressed proliferation, migration and invasion of PCa cells. Impaired cell proliferation is often caused by cell cycle arrest [23]. Consistently, we found that circDPP4 knockdown suppressed PCa cell cycle progression partly by inhibiting expression of cyclin D1, a well-known oncogene involved in cell cycle progression [24]. Besides, the oncogenic activity of circDPP4 was also verified in nude mouse xenograft model bearing PCa cells.

MiRNAs, a class of small non-coding RNAs, also play a critical role in cancer biology [25]. circRNA could act as ceRNA to regulate gene expression at the post-transcriptional level via sequestering miRNA [26, 27]. This characteristic is similar to that of long non-coding RNAs. Given that circDPP4 is abundant in the cytoplasm, we hypothesized that circDPP4 might serve as a ceRNA to sequester miRNAs in PCa as well. By performing bioinformatics analysis and dual-luciferase reporter assay, we revealed and validated that circDPP4 was able to bind with miR-195, which is widely reported as a tumor suppressor in PCa and other malignancies [28, 29]. In agreement with previous studies, we also showed that miR-195 expression was decreased and negatively correlated with circDPP4 expression in PCa tissues. Besides, through RNA-ISH analysis, we found these two non-coding RNAs co-localized in the cytoplasm of tumor tissues and tumor cell line, implying they are directly correlated.

MiRNAs act as critical signal transduction mediators by negative regulation of their target genes [30], thereby influencing cell fate and function. We then identified Cyclin D1 as a potential direct target of miR-195 in PCa. This interaction has been previously found in osteosarcoma [31] and cervical cancer [32]. Rescue experiments further showed that miR-195 diminished the oncogenic role of circDPP4 in PCa cells, indicating that circDPP4 might function as a ceRNA to sequester and reduce miR-195 activity, thus leading to the increase of cyclin D1 expression in PCa.

**Fig. 7:**
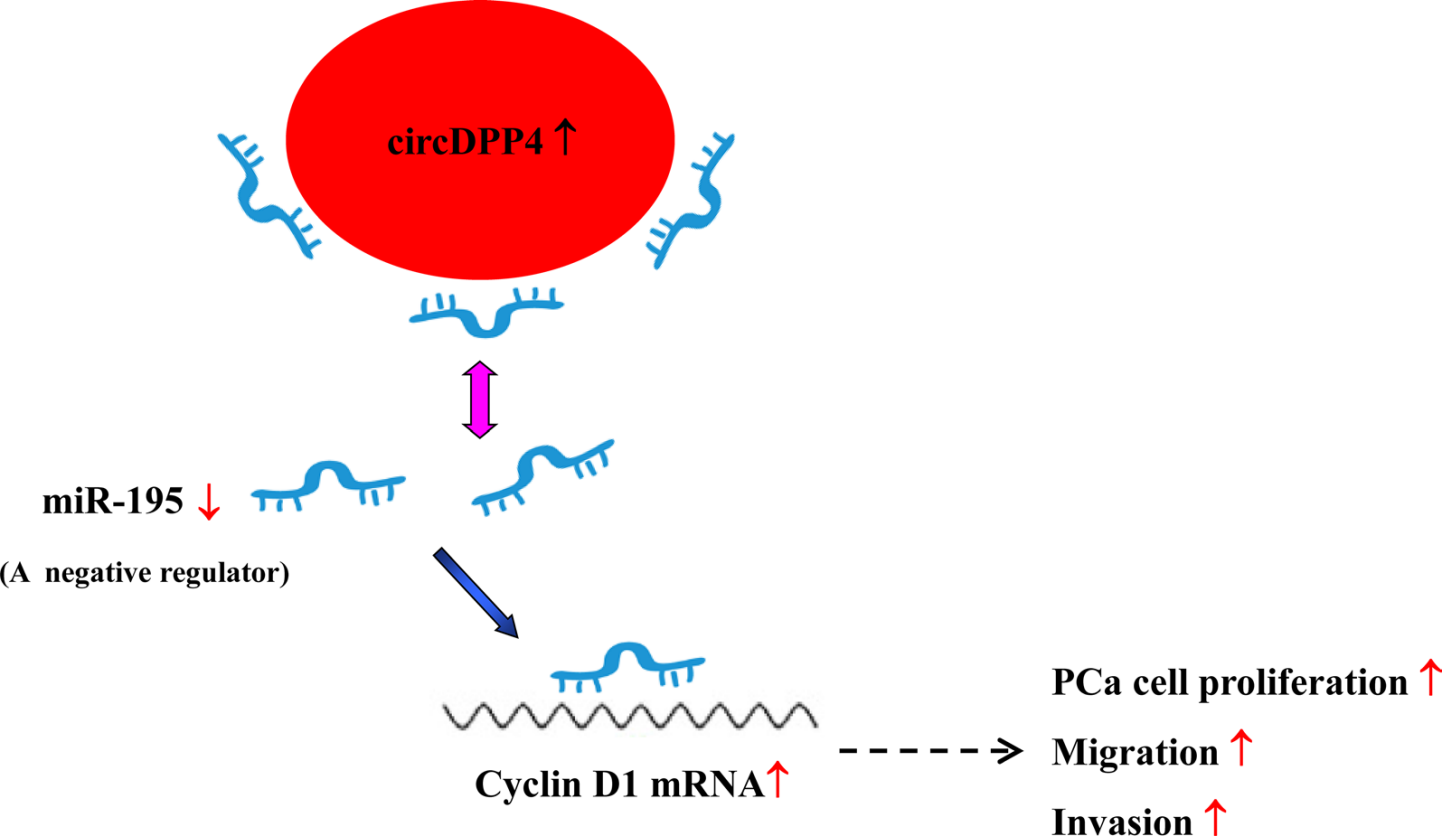
Schematic of circDPP4/miR-195/cyclin D1 axis in regulating proliferation, migration and invasion of PCa cells

In conclusion, this is the first report to reveal the expression level and biological function of circDPP4 in PCa. Our results demonstrated that circDPP4 was significantly upregulated in PCa tissues, and circDPP4 promoted cell growth, migration, invasion of PCa via sponging miR-195 to regulate cyclin D1 expression (Fig. 7). These findings suggested that circDPP4/miR-195/cyclin D1 axis might involve in the tumorigenesis and progression of PCa and could become a potential therapeutic target. Further research needs to be conducted to clarify the downstream pathway of circDPP4/miR-195/cyclin D1 axis.

## Supplementary Information

**Additional file 1: Figure S1.** Screening of differentially expressed circRNAs in prostate tissues and matched adjacent normal tissues using microarray analysis. (A) The heatmap; (B) the scatter plot; (C) the volcano plot of differentially expressed circRNAs.

**Additional file 2: Figure S2.** Cyclin D1 is a downstream target of miR-195 in PCa cells. (A) The predicted miR-195 binding sites in cyclin D1 mRNA 3′-UTR. (B) Dual-luciferase reporter assay performed to validate the direct binding relationship between miR-195 and cyclin D1 mRNA 3′-UTR. (C) Western blot analysis of cyclin D1 protein levels in PC3 and LNCaP cells after transfection. (D) The proliferation of PC3 and LNCaP cells after transfection detected by CCK-8 assay. *P < 0.05 vs. mimics control or inhibitor control-transfected cells.

**Additional file 3: Figure S3.** Co-localization of circDPP4 (green fluorescence) and miR-195 (red fluorescence) in the cytoplasm of PCa cells measured by RNA-fluorescence in situ hybridization (FISH). The nuclei were stained with DAPI (blue fluorescence).

**Additional file 4: Table S1.** 1021 differentially expressed circRNAs in PCa tissues were identified by microarray analysis.

**Additional file 5: Table S2.** Correlation between circDPP4 expression and clinicopathological characteristics of PCa patients.

**Additional file 6.** Representative melting curves and amplification curves of RT-qPCR reaction.

## Data Availability

The datasets used and/or analyzed during the current study are available from the corresponding author on reasonable request.

## Abbreviations

circRNAs: Circular RNAs
PCa: Prostate cancer
ceRNA: Competing endogenous RNA
