# Supplementary figures and images for: Circular RNA-DPP4 serves an oncogenic role in prostate cancer progression through regulating miR-195/cyclin D1 axis
Source: Cancer Cell Int. 2021 Jul 16;21:379. doi: 10.1186/s12935-021-02062-z (PMC8283928; doi:10.1186/s12935-021-02062-z)

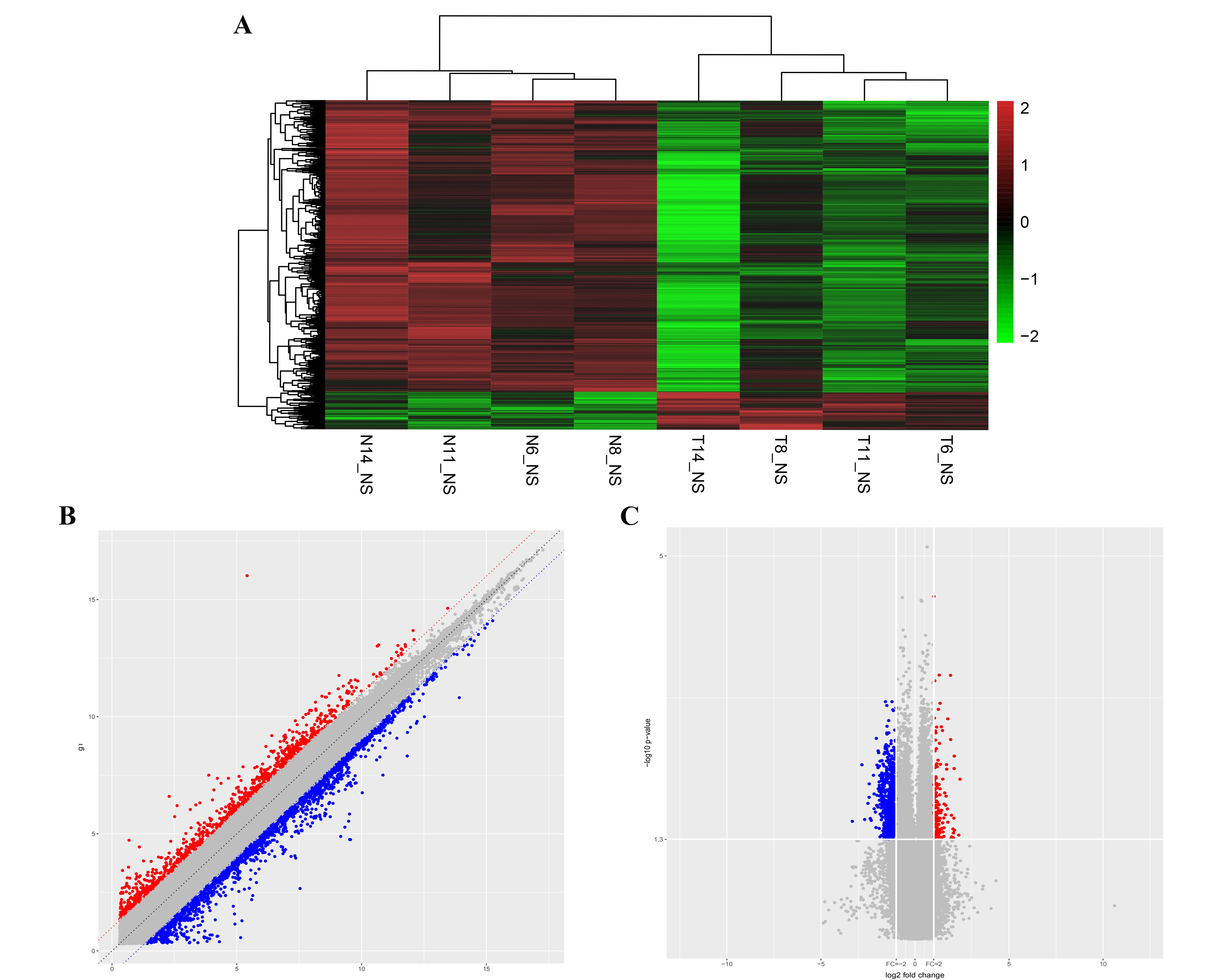

Supplement: Supplementary file 1 — Additional file 1: Figure S1. Screening of differentially expressed circRNAs in prostate tissues and matched adjacent normal tissues using microarray analysis. (A) The heatmap; (B) the scatter plot; (C) the volcano plot of differentially expressed circRNAs. [file 12935_2021_2062_MOESM1_ESM.jpg]

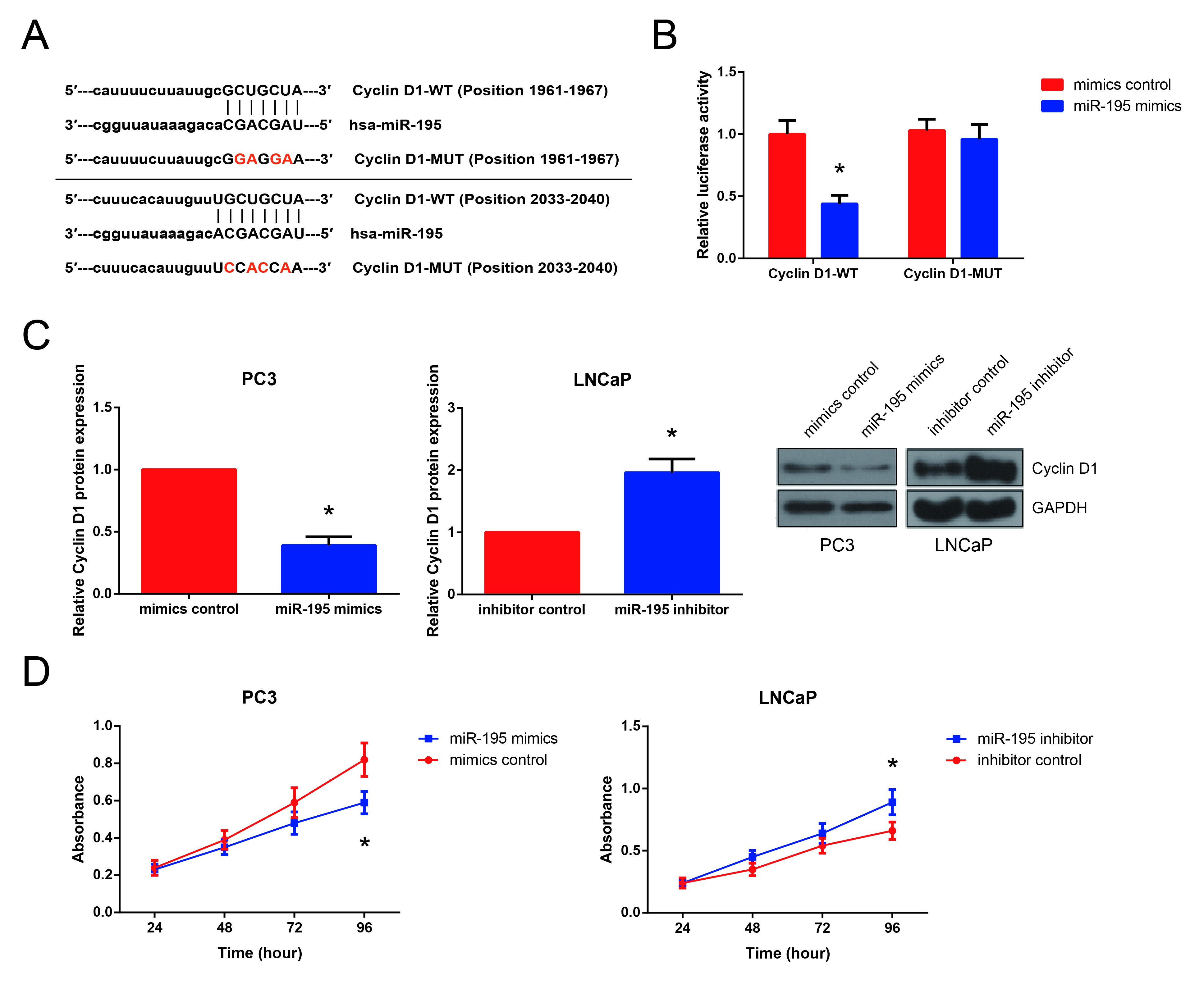

Supplement: Supplementary file 2 — Additional file 2: Figure S2. Cyclin D1 is a downstream target of miR-195 in PCa cells. (A) The predicted miR-195 binding sites in cyclin D1 mRNA 3′-UTR. (B) Dual-luciferase reporter assay performed to validate the direct binding relationship between miR-195 and cyclin D1 mRNA 3′-UTR. (C) Western blot analysis of cyclin D1 protein levels in PC3 and LNCaP cells after transfection. (D) The proliferation of PC3 and LNCaP cells after transfection detected by CCK-8 assay. *P < 0.05 vs. mimics control or inhibitor control-transfected cells. [file 12935_2021_2062_MOESM2_ESM.jpg]

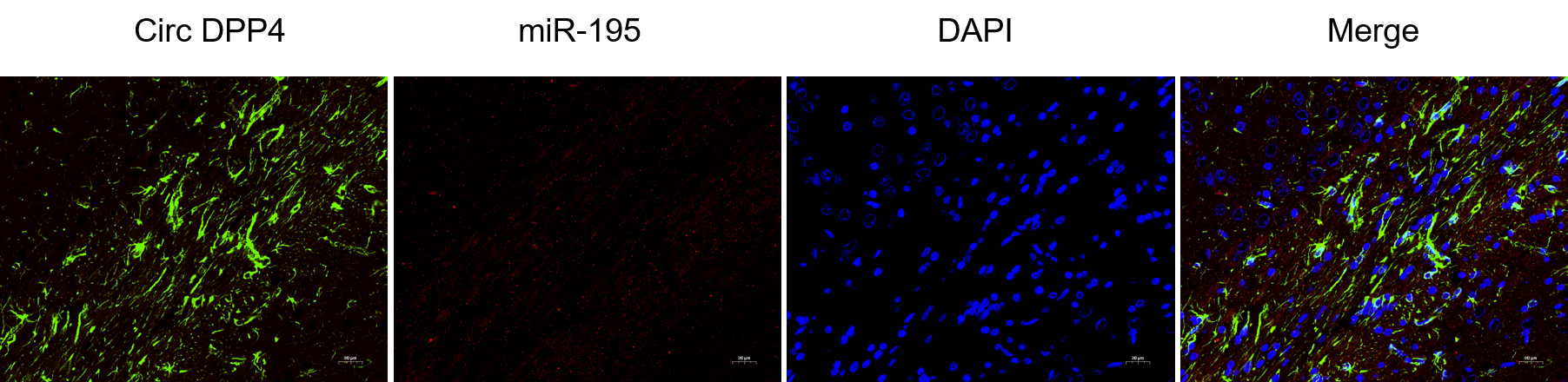

Supplement: Supplementary file 3 — Additional file 3: Figure S3. Co-localization of circDPP4 (green fluorescence) and miR-195 (red fluorescence) in the cytoplasm of PCa cells measured by RNA-fluorescence in situ hybridization (FISH). The nuclei were stained with DAPI (blue fluorescence). [file 12935_2021_2062_MOESM3_ESM.jpg]
