# Supplementary material for: Circular RNA-DPP4 serves an oncogenic role in prostate cancer progression through regulating miR-195/cyclin D1 axis
Source: Cancer Cell Int. 2021 Jul 16;21:379. doi: 10.1186/s12935-021-02062-z (PMC8283928; doi:10.1186/s12935-021-02062-z)
Supplement: Supplementary file 4 — Additional file 4: Table S1. 1021 differentially expressed circRNAs in PCa tissues were identified by microarray analysis. [file 12935_2021_2062_MOESM4_ESM.docx]

**Table S1. The Primer sequences used in RT-qPCR**

| **Gene** | **Primer Sequences** |
| --- | --- |
| crircDPP4 | Forward: 5’-AATGAGAGGGAAGAGCGGAG-3’ |
|  | Reverse: 5’-ACATCCACGTCCTTTCCCAT-3’ |
| GAPDH | Forward: 5’- CACATCGCTCAGACACCATG -3’ |
|  | Reverse: 5’- TGACGGTGCCATGGAATTTG-3’ |
| miR-195 | Forward: 5’- TAGCAGCACAGAAATATTGGC -3’  Reverse: 5’- TGCTGTGCCAGCTGCAGTCG-3’ |
| U6 | Forward: 5’-CTCGCTTCGCCAGCACA-3’  Reverse: 5’- TAACGCTTCACGAATTTGCGT-3’ |
| Cyclin D1 | Forward: 5’-TGAGGTGAGGGCCAACTTAG-3’  Reverse: 5’- CCCTTCCCAACCCTCATAGAC-3’ |
