# Supplementary material for: Circular RNA-DPP4 serves an oncogenic role in prostate cancer progression through regulating miR-195/cyclin D1 axis
Source: Cancer Cell Int. 2021 Jul 16;21:379. doi: 10.1186/s12935-021-02062-z (PMC8283928; doi:10.1186/s12935-021-02062-z)
Supplement: Supplementary file 5 — Additional file 5: Table S2. Correlation between circDPP4 expression and clinicopathological characteristics of PCa patients. [file 12935_2021_2062_MOESM5_ESM.doc]

**Table S2** Correlation between circDPP4 expression and clinicopathological characteristics of PCa patients.

| Characteristics | Total number  (n=104) | circDPP4 expression | | *P* value |
| --- | --- | --- | --- | --- |
|  |  | Low (n=52) | High (n=52) |  |
| Age (years) |  |  |  | 0.239 |
| ≥ 70 | 54 | 24 | 30 |  |
| < 70 | 50 | 28 | 22 |  |
| Gleason score |  |  |  | 0.015* |
| ≥ 8 | 66 | 27 | 39 |  |
| < 8 | 38 | 25 | 13 |  |
| Preoperative PSA (ng/ml) |  |  |  | 0.163 |
| ≥ 20 | 80 | 37 | 43 |  |
| < 20 | 24 | 15 | 9 |  |
| Clinical stage |  |  |  | 0.026* |
| T2A-T2C | 65 | 38 | 27 |  |
| T3A-T4 | 39 | 14 | 25 |  |
| Lymph node metastasis |  |  |  | 0.110 |
| Present | 62 | 27 | 35 |  |
| Absent | 42 | 25 | 17 |  |

* Comparison of clinicopathological characteristics between PCa patients with high circDPP4 expression and low circDPP4 expression.
