# Supplementary material for: Circular RNA-DPP4 serves an oncogenic role in prostate cancer progression through regulating miR-195/cyclin D1 axis
Source: Cancer Cell Int. 2021 Jul 16;21:379. doi: 10.1186/s12935-021-02062-z (PMC8283928; doi:10.1186/s12935-021-02062-z)

**Additional file 6**

Representative melting curves and amplification curves of RT-qPCR reaction

GAPDH


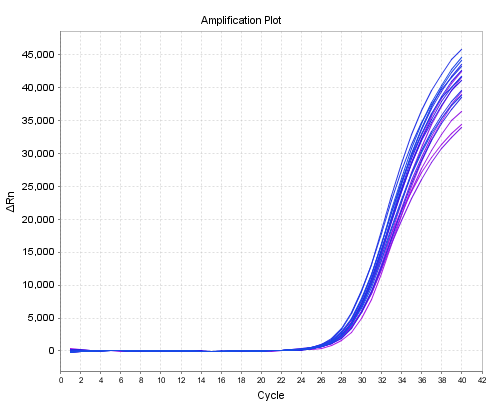

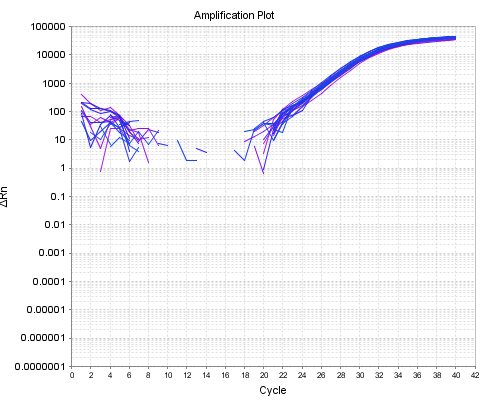

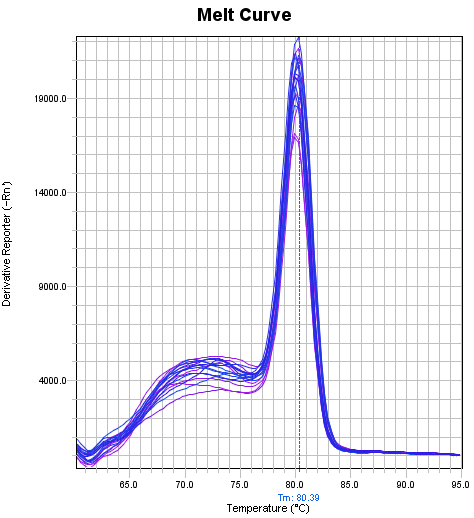


crircDPP4


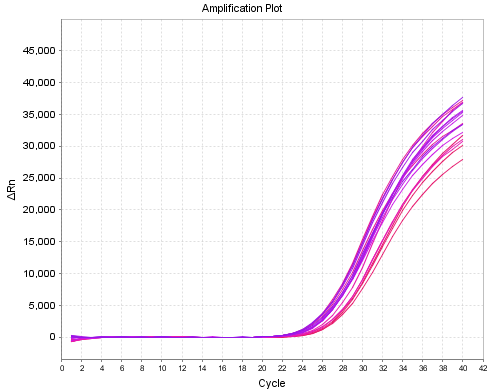

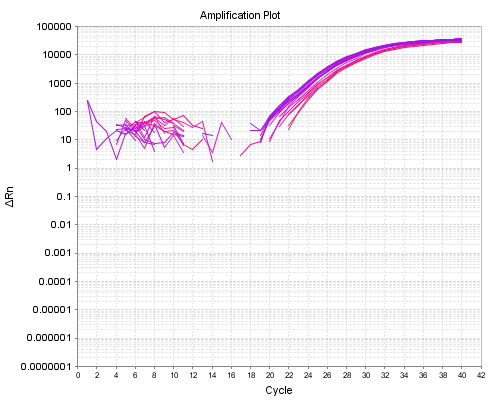

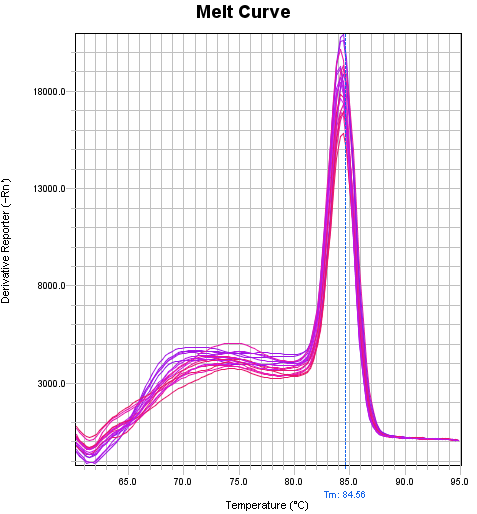

Supplement: Supplementary file 6 — Additional file 6. Representative melting curves and amplification curves of RT-qPCR reaction. [file 12935_2021_2062_MOESM6_ESM.doc]
